# Social support and economic conditions among older migrants in India: do distance, duration, and streams of migration play a role in later life?

**DOI:** 10.1186/s12889-024-19165-7

**Published:** 2024-07-10

**Authors:** Vasim Ahamad, Ram Babu Bhagat, Manisha, Sanjay Kumar Pal

**Affiliations:** 1 Department of Migration and Urban Studies, International Institute for Population Sciences, Govandi Station Road, Deonar, Mumbai, 400088 India; 2 Department of Fertility & Social Demography, International Institute for Population Sciences, Govandi Station Road, Deonar, Mumbai, 400088 India

**Keywords:** Social support, Economic condition, Ageing, Migration, Older migrants

## Abstract

**Background:**

Being older and having a migrant feature might cause a double risk of vulnerability in poor economic, social support, and health status at the place of destination. This study examines the association of migration on the social support and economic condition of older persons in India.

**Methods:**

Longitudinal Ageing Study in India (LASI) wave-I (2017–2018) data with total samples of 66,156 older adults aged 45 + with 30,869 and 35,287 male and female samples, respectively, used in this study. Descriptive and bivariate analyses have been performed to examine the pattern of older migrants, and multinomial logistic regression analysis has been used to establish the associations between migration, social support, and economic condition.

**Results:**

Over half (57.5%) of the population aged 45 + in India had migrant characteristics; 80% migrated before 25 years. Of all migrants, about 90% migrated within one state (Intrastate), and 9% migrated to another (Interstate). The association between social support and migration by distance and the adjusted result showed that immigrants were less likely to have medium [RRR = 0.56 (CI; 0.46–0.68)] and high [RRR = 0.39 (CI; 0.30–0.50)] social support. The interstate migrants were also less likely to have high [RRR = 0.90 (CI; 0.83–0.98)] social support. The migrants with 0–9 years of duration were less likely to have high social support, and the urban to rural stream migrants were more likely to have high social support. The association between economic status and migration by distance and the adjusted result showed that more affluent immigrants were likelier to have [RRR = 1.41 (CI; 1.14–1.73)] better economic conditions than affluent non-migrants. Migrants with 0–9-year duration and urban to rural stream were found to be likelier to have better economic conditions.

**Conclusions:**

The findings of this study suggest that distance, duration, and migration stream have a significant association with social support and economic conditions in later life. In exploring migration’s effect on social and economic status, policymakers should prioritize migrants in their agenda to maintain socio-economic and social support for older persons in India to achieve the sustainable goal of active and healthy ageing.

## Background

With the increase in the older population due to increased life expectancy and fertility decline [1], the share of older migrants also increases in many developed and developing countries, and migration and ageing have become two intertwined research topics [2]. While ageing is an achievement in human society in terms of medical advancements and economic and social development over diseases, injuries, and early deaths that have limited human life spans throughout history [3], it has put before us enormous challenges [4] in terms of providing social security and access to health care to the older adults. Migration plays an essential role in economic living conditions and individual well-being and supports care in later life [5, 6]. It is well known that increasing age is an independent risk factor for developing non-communicable diseases (NCDs) such as cardiovascular disease, cancer, diabetes, and dementia [1, 7]. Social interactions must be maintained with increasing age, as good social functioning is associated with improved self-efficacy [8, 9], reduced risk of depression [10, 11], and a reduced risk of all-cause mortality [12]. World Health Organisation (WHO) identifies social support as a key determinant of active and healthy ageing [13].

Social support refers to individuals’ relationships with others, including formal and informal ones, such as family members, relatives, friends, peers, or community organizations [14]. In times of need or crisis, social support gives individuals a broader focus and positive self-image, which enhances the quality of life, particularly in old age. Social support is closely linked to positive health and psychological well-being [15] and encompasses more than physical presence and social care. Economic conditions are essential for older people to have a positive sense of well-being as they directly affect their everyday lives and social prestige [16]. Low economic status is also associated with poor health, especially among older adults [17]. A large and growing number of studies from both developing and developed countries suggest solid and positive associations between economic indicators and longevity, nutrition, and healthcare utilization [18, 19]. The economic well-being of households is the key determinant of older people’s health. Without a robust and universal social security system, the low coverage of old-age pensions, a large share of employment in the informal sector, early retirement from formal employment, and increasing health expenditure, older person households in India are economically vulnerable and prone to financial shock [16]. Furthermore, increasing urbanization, rural-urban migration, and modernization have led to several socioeconomic changes, including changes in the structure of families and living arrangements. Migration plays a role in the social support and economic status of older persons at the destination place [6].

Migration is the temporary or permanent movement of a person away from their usual residence, either across an international border or within a state of a country [20]. In the migration process, the young population experiences greater mobility than other ages [21–23], so the researcher mainly focuses on the young population or labour force movement in migration studies. More research is needed to explore the intersection between ageing and migration [2]. Migration and ageing are two of the foremost contemporary phenomena that are challenging for modern societies. They are separate dynamic phenomena at first glance, but there are multiple intersections between the demographic ageing of the population and the increasing number of people migrating [24]. There are at least three ways in which old age and migration cross each other paths: (1) People usually migrate at a relatively young age and have grown old at the place of destination (ageing in place of destination), (2) Older people migrate when they become older because of retirement, family rejoining, and institutional needs (Older migration), and (3) The out-migration of young people, mainly from rural to urban, resulting in older people being left behind without children to look after them (Left behind older persons). Migration profoundly affects older people’s socioeconomic and health well-being and care in all migration-ageing intersection cases [6, 25]. Much of the extant literature focuses on the economic performance of younger and recently arrived migrants [26–28]. However, very little literature exists on social support and economic status among older migrants, suggesting that migration negatively affects socioeconomic well-being [6–29]. Positive association with economic conditions, but factors of the migration process are also associated with it [30]. Due to a lack of data set and less interest by researchers in migration studies with an ageing perspective, the direct impact of migration on individual social and economic status has yet to be explored much. How is migration associated with social support and economic status in later life? Distance, duration, and migration stream have a role in older persons’ socioeconomic conditions. This question must be examined in the Indian context because the ageing population has continuously increased in India over the last decades [31, 32], which is why the share of older migrants has also increased. According to the census of India 2011, 103 million people were 60 and above age, which increased from 5.6% in 1961 to 8.6% in 2011 to the total population of India, and it will reach 20% of the total population in 2050, according to UN projection [33]. According to the 2011 census, 53 million people aged 60 + were migrants, 51% of the total elder population [34], meaning half of India’s older population were migrants. From the 2001 census to 2011, the number of older migrants changed from 34.6 million to 53.8 million, a 55.2% increase between the census periods [34].

This paper focuses on the level and pattern of older adult’s migration and the distribution of social support and economic status among older migrants in India. Moreover, it examines social support and economic association with migration. The paper argued that the migration pattern (distance, duration, and stream) affects older adults’ social support and economic status in later life, and migrants have less social support and higher economic status at destination places than non-migrants. For this, the economic status is assessed using an economic measure indicator, monthly per capita consumption expenditure (MPCE) [35], and defined as poor, middle, and rich economic status categories. Social support is measured according to studies [36]; survey questions based on participation in social activities were assessed to generate this variable, and social support is defined as no social support, low social support, and high social support.

## Data source and methodology

### Data source

A cross-sectional study design was adopted for this study. Data for the analysis were drawn from the Longitudinal Ageing Study in India (LASI), wave one, collected from 2017 to 18. It is a nationally representative survey of 73,396 individuals, 31,135 male and female, 42,261 aged 45 years and above, and their spouses (regardless of age) across all states and union territories of India. The survey’s main objective was to study the health status and socioeconomic well-being of older adults in India. The LASI adopted a multistage stratified area probability cluster sampling design to arrive at the eventual observation units: older adults age 45 and above and their spouses, irrespective of age. Within each state, LASI Wave 1 adopted a three-stage sampling design in rural areas and a four-stage sampling design in urban areas. In each state/UT, the first stage involved the selection of Primary Sampling Units (PSUs), that is, sub-districts (Tehsils/Talukas), and the second stage involved the selection of villages in rural areas and wards in urban areas in the selected PSUs. In rural areas, households were selected from selected villages in the third stage. However, sampling in urban areas involved an additional stage. Specifically, one Census Enumeration Block (CEB) was randomly selected in the third stage in each urban area. In the fourth stage, households were selected from this selected CEB (LASI Report) [35]. The listed HHs from the selected CEB were used as the sampling frame for HH selection. The present study was conducted on respondents aged 45 years and above. The final sample size was 66,156 older adults selected after excluding individuals below age 45 (6,790), missing values (222), and return migrants (228). The migrants are classified based on the place of last residence (POLR) and place of enumeration for this study. Return migrants are defined as persons whose POLR is different from their current place, and their current place is their place of birth (POB). This study mainly focuses on older migrants at the destination and compares their socioeconomic condition with native-born or local populations in India.

### Study variables

#### Dependent variable

The outcome variables are social support and economic status. The social support defined according to studies [36], survey questions based on participation in social activities were assessed to generate this variable. The activities of individuals included receiving financial support, visiting relatives/ friends, attending cultural performances/ shows/ cinema, attending religious functions/ events, and attending community/ political/organization group meetings (Cronbach’s alpha = 0.62). They were recoded into yes and no (“yes” as 1 = at least once in a month, and “no” as 0 = rarely or never). Scores of 0 to 9 are categorized into three categories (Low, medium, and high social support). In the LASI survey, data on consumption expenditure are collected using the abridged version of the National Sample Survey (NSS) consumption schedule. Sets of 11 and 29 questions on the expenditures on food and non-food items, respectively, were used to canvas the sample households. Food expenditure was collected based on a reference period of seven days, and non-food expenditure was collected based on reference periods of 30 days and 365 days. Food and non-food expenditures have been standardized to the 30-day reference period. The monthly per capita consumption expenditure (MPCE) is computed, used as the summary measure of consumption, and used as the MPCE quantile in five categories. We used this MPCE quantile for the analysis as poor, middle, and rich with a mean of 1495, 2496, and 4771 rupees, respectively.

#### Independent variables

##### Migration status

Persons are classified as migrants based on the question “Place of last residence (POLR).” According to this, if a person’s place of last residence is different from the current place, then the person is considered a migrant; otherwise, non-migrant in this study; the duration of migration is classified with the question “How many years have you continuously lived in this place” If the person answers since birth, then the persons consider a non-migrant. Otherwise, migrants and calculate the migration duration. The migration pattern of older adults examined with migration duration, which is categorized as 0 to 9 years, 10 to 24 years, and 25 and above years; migration distance (Intrastate, Interstate, and Immigrants); migration stream (Rural to Rural, Rural to Urban, Urban to Rural and Urban to Urban**)**, and age at migration (0–14 years, 15–44 years, 45–59 years, and 60 and above years).

The migration duration has been stratified into three categories (0–9, 10–24, and 25 + years). The first category of duration (0–9 years) of migration has been chosen to observe the proportion of migration within decades (shorter duration); this is also referred to as intercensal duration (the duration between two censuses) of migration [37]. Afterward, 10–24 and 25 + years of migration have been categorized to see the proportion of migrated persons between medium (10–24 years) and long (25 + years) migration duration.

According to the literature, migration distance is categorized based on administrative boundaries, assuming that migration within a state constitutes a short distance. In contrast, migration from one state to another and from one country to another is considered to be long-distance. The rationale for including this variable in the study is to examine social support and economic conditions among migrants with different migration distances. However, it is acknowledged that the categorization of distance based on state boundaries has limitations, particularly in accurately reflecting the actual distances covered. As mentioned, the distance between adjacent states may be shorter than the within-state distance for some migrants [37, 38].

The other independent variables are age (45–59, 60–69, 70–79, and 80 + years**);** sex (male and female); place of residence (rural and urban); marital status (currently married, widowhood, and others), religion (Hindu, Muslim, and others); Caste (Scheduled caste, Scheduled tribes, Other Backward Class and Others) education (No education, below primary, primary, above primary, secondary and higher, and graduate and above); Living arrangement (living alone, living with spouse and children, living with spouse and others, living with children and others and living with others); currently working (working, not working and never worked) and Regions (North, East, Northeast, West, and South).

### Statistical analysis

The study participants’ general characteristics and distribution were determined using descriptive analysis. The preliminary study used descriptive statistics and univariate and bivariate analysis to examine migration levels, patterns, and other independent variables’ characteristics with social support and monthly per capita consumption expenditure. Chi-square tests and *p*-values have been used to see the independence of the variables and the significance of the output results, respectively. Aside from that, the findings of the association of social support and monthly per capita consumption expenditure with migration status and other independent variables were carved out using multinomial logistic regression analysis.

A multinomial logistic regression model can be written as follows:

$$Logit \left(pi\right)=ln\left(\frac{\text{p}\text{i}}{1-pi}\right)={\beta }_{0}+{{\beta }}_{1}{\text{x}}_{1}+{{\beta }}_{2} {\text{x}}_{2}+\dots +{{\beta }}_{k} {\text{x}}_{k}$$

Where ${\beta }_{0}$is the intercept, ${{\beta }}_{1}, {{\beta }}_{2},\dots .\dots \dots ,{{\beta }}_{k}$are the regression coefficients indicating the relative effect of a particular explanatory variable on the outcome, ${\text{x}}_{1}, {\text{x}}_{2},\dots .\dots \dots ,{\text{x}}_{k}$, are the control variables [39].

For the multicollinearity among the independent variables, we checked the collinearity using Spearman correlation coefficients. We found no collinearity among independent variables except the migration stream with the place of residence (*r* = 0.76). Therefore, we have dropped the residence variable from the model-III of Tables 3 and 4 for social support and economic conditions of migrants, respectively. Furthermore, variance inflation factors (VIFs) were also used to check for multicollinearity with the exposure migration variables distance, duration, stream, and age at migration variables and other independent variables for each model separately. We found an average VIF (1.28) for each type of migration pattern in the models, which suggests that models have no collinearity among the study variables. The statistical package STATA for Windows version 16 was used for all statistical analyses [40]. The proper individual-level sampling weights were used to make the results representative.

## Results

### Migration pattern of older adults in India

**Fig. 1:**
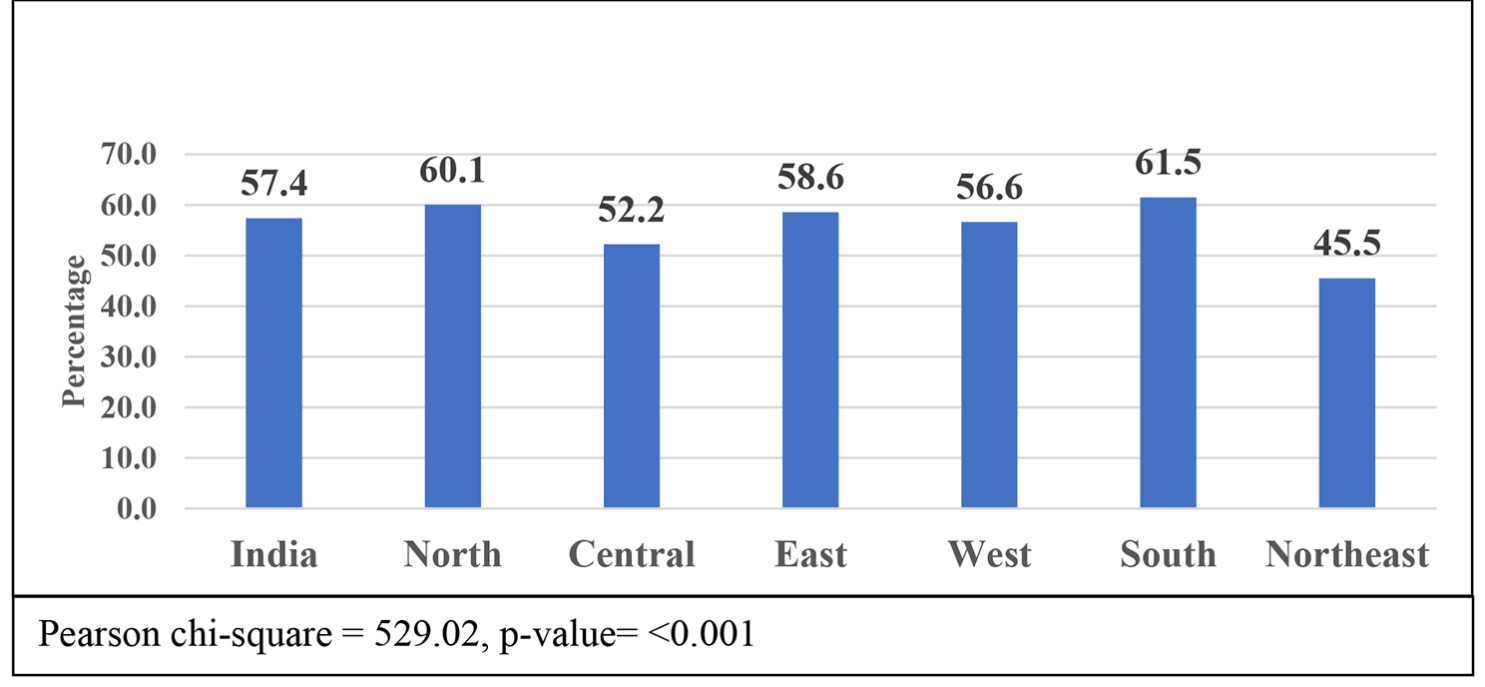
Migration proportion of older adults (45+) in India and its regions, LASI Wave-I (2017-18)

Figure 1 shows the migration proportion of older adults aged 45 and above in India and its regions. The figure indicates that 57.4% of older adults were migrants in India. Furthermore, the proportion of region-wise migrants of older adults showed that the highest concentration of migrants was from the South (61.5%) and North (60.1%) regions, and the lowest concentration of migrants was from the Northeast (45.5%) and Central (52.2%) regions

Table 1 depicts the level and pattern of older adults’ migration, and it shows that among total migration, females have migrated more (80.3%) than males (30.4%), only 6.4% of migrants migrated in the last nine years, and 80.1% of migration occurred before twenty-five or more years. Intrastate migration is dominant in older migration in India; It covers a total of 89.6% of total older migration, and only 9.2% and 1.3% of migration occurred in interstate and international (immigrants) migration, respectively. Most migrants migrated through the rural-to-rural stream 61.0%, then rural-to-urban (21.2%), and urban-to-urban (15.1%). About 72% of older adults migrated at their childhood (0–14 years) age, 18% migrated at their working age (15–44 years), 7% migrated at their pre-retirement age (45–59 years), and 4.4% of older migrants migrated at their age 60 and above. The age at migration varies by sex and residence; the migration at age 60 and above is higher in urban (5.1%) than in rural areas (1.6%), and male migrants (4.4%) show higher percentage than female migrants (2.4%).

**Table 1:** Level and pattern of older adults (45 + age) Migration in India, LASI Wave-I (2017-18)

Migration Pattern	Percentage					Total number
	Male	Female	Total	Rural	Urban	
**Distribution of Migration**						
Migrants	30.4	80.3	57.4	53.4	66.1	37,211
Non-migrants	69.6	19.7	42.6	46.6	34.0	28,945
*p-value*	*< 0.001*			*< 0.001*		
**Total**	**100.0**	**100.0**	**100.0**	**100.0**	**100.0**	**66,156**
**Duration of Migration**						
0–9 year	12.7	4.4	6.4	3.5	11.5	2,699
10-24-year	19.9	11.5	13.6	8.1	23.1	5,924
25 + year	67.5	84.1	80.1	88.4	65.4	28,588
*p-value*	*< 0.001*			*< 0.001*		
**Distance of Migration**						
Intra-state	85.2	91.0	89.6	94.6	80.7	31,046
Interstate	12.7	8.0	9.2	4.0	18.2	5,604
Immigrants	2.1	1.0	1.3	1.4	1.1	561
*p-value*	*< 0.001*			*< 0.001*		
**Stream of Migration**						
Rural-Rural	46.2	65.8	61.0	95.8	0.0	20,651
Rural-Urban	28.1	19.0	21.2	0.0	58.5	9,479
Urban-Rural	2.5	2.8	2.7	4.3	0.0	1,012
Urban-Urban	23.2	12.5	15.1	0.0	41.6	5,509
*p-value*	*< 0.001*			*< 0.001*		
**Age at migration**						
0–14	60.7	75.3	71.7	80.8	55.8	25,126
15–44	21.0	17.4	18.3	13.6	26.4	8,077
45–59	14.0	4.9	7.1	4.0	12.7	2,848
60 and above	4.4	2.4	2.9	1.6	5.1	1,160
*p-value*	*< 0.001*			*< 0.001*		
**Total**	**100.0**	**100.0**	**100.0**	**100.0**	**100.0**	**37,211**

### Social support and economic condition of older adults in India

**Fig. 2:**
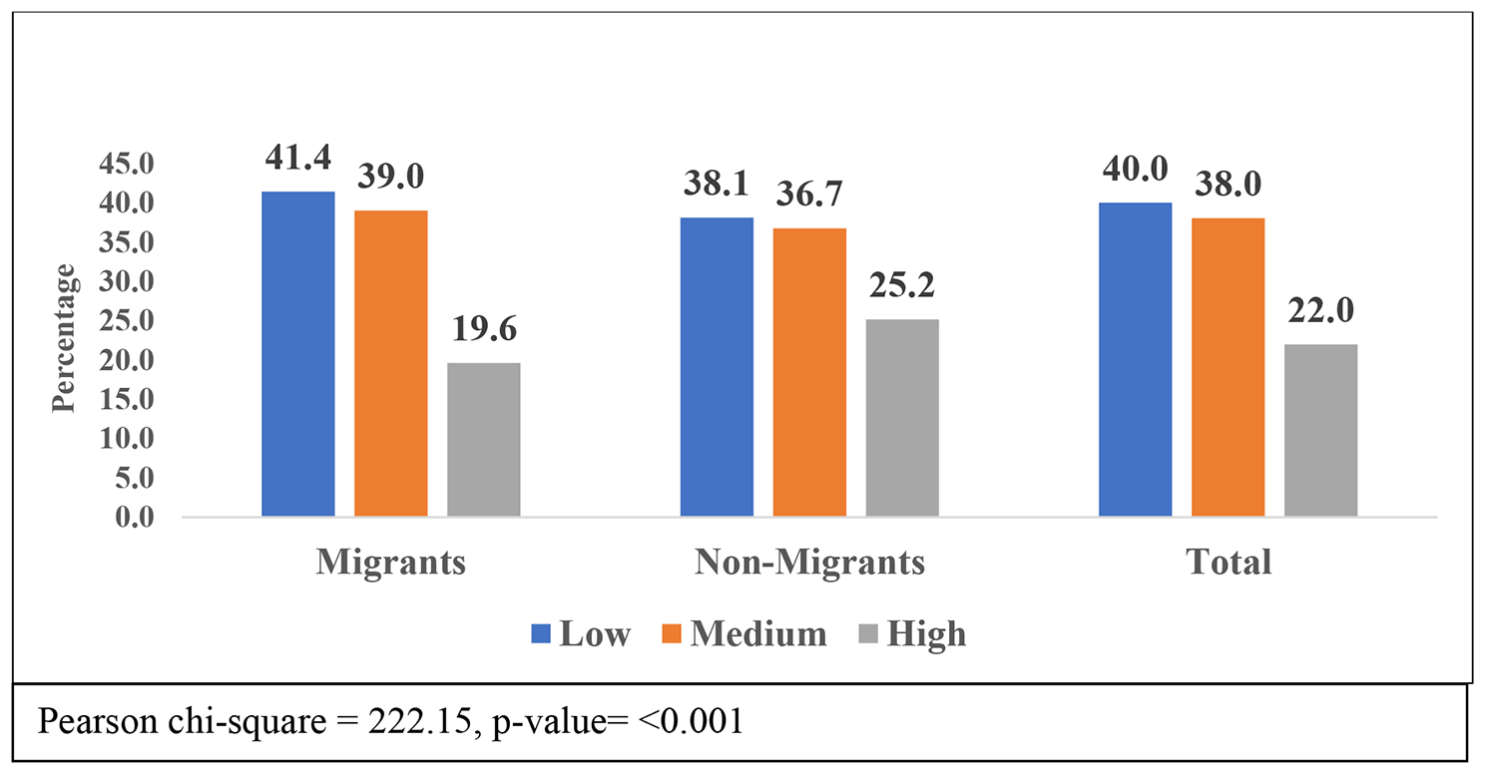
Social Support of older adult with migration status in India, LASI Wave-I (2017-18)

Figure 2 shows the social support status with migration. The figure indicates that 40% of total older adults had low social support in India, 38% had medium, and only 22% had higher social support. Migrant older adults had a higher percentage of low social support (41.4%) than non-migrants (38.1%) older adults. Among high social support, the non-migrants (25.2%) were more dominant than migrants (19.6%).

**Fig. 3:**
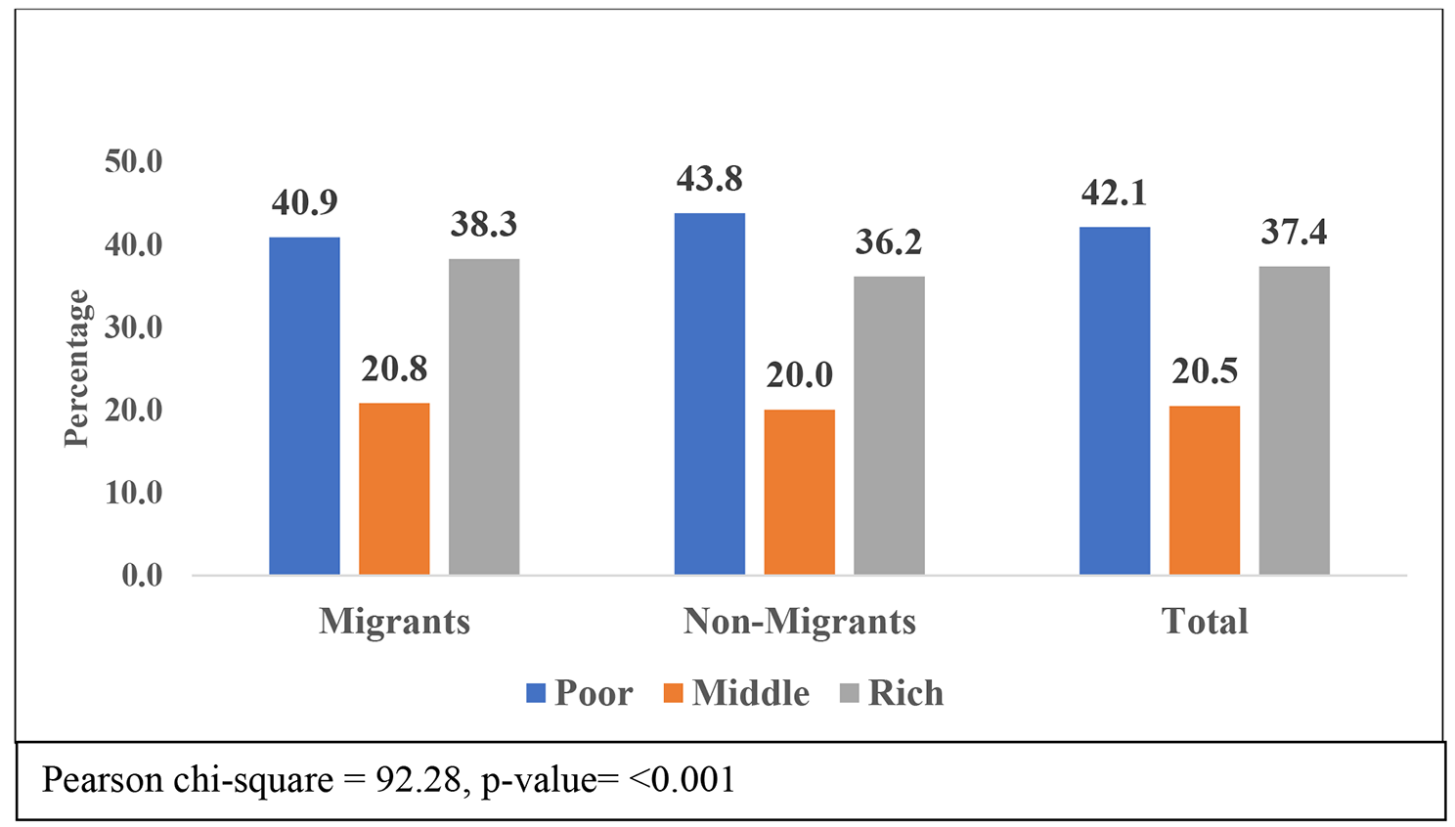
Economic condition of older adult with migration status in India,, LASI Wave-I (2017-18)

Figure 3 shows the monthly per capita expenditure as an economic status of older adults with migration in India. This figure showed that 42.1% of older adults had poor economic conditions in India, and 37.4% had rich economic conditions. The proportion of poor non-migrants (43.8%) was higher than poor migrants (40.9%), and the proportion of rich economic conditions migrants (38.3%) was higher than non-migrants (36.2%)

Table 2 depicts the distribution of older adults’ social support and economic conditions with all demographic, socioeconomic, and migration patterns. The result showed a statistically significant association between socio-demographic and migration patterns with social support and economic conditions. The results indicate that as older individuals age, the prevalence of low social support and poor economic conditions also continues to rise. Furthermore, the results demonstrate that more than half of the population (57%) aged 80 and above experience lower social support, while approximately 46% face challenging low economic conditions. In the case of sex, a higher proportion of male older adults had high social support (26.9%) and rich economic conditions (37.8%) than females with high social support (17.8%) and rich economic conditions (37%), whereas for the low and medium social support and poor and middle economic conditions male were had lower levels of low and medium social support and poor and middle economic conditions than females. The rural population showed a lower proportion of high social support and rich economic conditions than the urban population. Higher education showed a higher proportion of medium social support and rich economic conditions. In the case of caste, a higher proportion of low social support (44%) and poor economic conditions (49.6%) were observed in the older adults belonging to scheduled caste. The religion-wise results showed that older adults belonging to Hindus and Muslims had a higher proportion of low social support (40.9%) and poor economic conditions (43.7%), respectively. Older adults living with spouses and children had higher levels of high social support, whereas older adults living alone had higher levels of low social support. However, older adults living alone (49.2%) and living with a spouse and others (51.6%) had higher levels of high economic conditions.

**Table 2:** Descriptive table of socioeconomic and migration variables with social support and economic status of older adults in India, LASI Wave-I (2017-18)

Variables	Social Support			Economic Conditions			Total Number
	Low	Medium	High	Poor	Middle	Rich	
**Age** ^***^							
45–59	36.0	39.1	24.9	40.8	20.0	39.2	34,514
60–69	40.3	38.5	21.3	43.5	20.4	36.1	19,076
70–79	46.5	36.0	17.5	42.5	22.0	35.4	9,161
80+	56.7	31.6	11.7	45.9	20.7	33.4	3,405
**Sex** ^***^							
Male	36.1	37.0	26.9	41.7	20.5	37.8	30,869
Female	43.3	38.9	17.8	42.5	20.5	37.0	35,287
**Residence** ^***^							
Rural	42.9	36.4	20.7	42.6	20.9	36.6	42,957
Urban	33.6	41.7	24.7	41.2	19.7	39.1	23,199
**Marital status** ^***^							
Currently married	37.2	38.4	24.4	40.8	20.8	38.4	49,269
Widowed	48.2	36.9	14.9	46.4	19.7	33.9	14,637
Others	43.0	38.5	18.5	41.7	18.1	40.3	2,250
**Education** ^***^							
No-education	47.0	36.9	16.1	49.3	20.4	30.4	31,154
Below Primary	37.4	37.9	24.7	44.5	20.2	35.3	7,545
Primary	34.5	40.0	25.5	39.4	21.2	39.4	8,701
Above primary	35.0	38.8	26.3	37.3	21.2	41.5	6,298
Secondary and Higher	31.0	38.7	30.3	27.2	21.5	51.3	8,740
Graduate and above	21.3	41.3	37.4	19.1	17.5	63.4	3,718
**Caste status** ^***^							
Scheduled caste	44.1	36.7	19.2	49.6	20.4	30.0	10,966
Scheduled tribe	40.7	36.7	22.6	58.6	17.0	24.5	11,674
Other backward class	39.3	39.1	21.7	41.0	21.1	38.0	25,111
Others	38.0	37.6	24.4	33.5	20.7	45.8	18,405
**Religion** ^***^							
Hindu	40.9	37.8	21.3	42.3	20.8	36.9	48,402
Muslim	38.1	38.6	23.3	43.7	19.4	36.9	7,744
Others	31.6	40.5	27.9	37.4	18.0	44.6	10,010
**Working status** ^***^							
Currently working	36.0	37.0	27.1	43.1	20.3	36.7	30,301
Not working	41.8	36.9	21.3	43.0	20.9	36.1	17,361
Never worked	45.2	41.1	13.7	39.6	20.5	39.9	18,494
**Living Arrangement** ^***^							
Living alone	62.7	28.2	9.1	31.2	19.7	49.2	2,303
Living with spouse and others	44.0	35.9	20.1	27.9	20.5	51.6	10,369
Living with spouse and children	35.2	39.1	25.8	44.8	21.0	34.3	37,990
Living with children and others	44.5	39.1	16.4	47.5	19.8	32.7	12,542
Living with others	47.0	36.6	16.4	48.4	18.8	32.8	2,952
**Regions** ^***^							
North	44.9	37.3	17.8	31.9	19.6	48.5	11,870
Central	44.2	37.4	18.4	50.2	19.5	30.3	8,878
East	44.7	40.3	15.0	49.8	21.4	28.9	11,512
West	29.0	35.0	36.0	42.7	20.4	36.8	8,797
South	39.0	39.1	21.9	32.7	21.1	46.2	15,592
Northeast	25.4	36.1	38.6	41.9	20.0	38.2	9,507
**Duration of Migration** ^***^							
0–9 year	39.6	35.4	25.0	28.7	24.1	47.2	2,699
10–24 year	34.9	42.7	22.4	34.9	19.6	45.5	5,924
25 + year	42.6	38.7	18.7	42.9	20.8	36.3	28,588
**Distance of Migration** ^***^							
Intrastate	41.9	38.5	19.6	41.2	21.1	37.7	31,046
Interstate	34.6	44.8	20.6	37.2	18.3	44.5	5,604
Immigrants	55.2	33.7	11.0	43.3	23.3	33.4	561
**Stream of Migration** ^***^							
Rural-Rural	45.2	37.1	17.8	42.8	21.0	36.2	20,651
Rural-Urban	35.7	42.3	22.0	43.9	18.8	37.3	9,479
Urban-Rural	38.8	38.2	23.0	25.9	23.4	50.6	1,012
Urban-Urban	33.3	42.8	23.9	31.4	22.5	46.1	5,509
**Age at migration** ^***^							
0–14	43.3	38.4	18.3	43.4	21.0	35.6	25,126
15–44	34.1	42.9	23.0	36.0	19.6	44.4	8,077
45–59	41.3	34.3	24.4	29.6	23.0	47.5	2,848
60 and above	41.4	40.6	18.1	36.3	19.8	43.9	1,160

Note: *** *p* = < 0.001, **p = < 0.05, & * *p* = < 0.1

Afterward, coming to the migration patterns with older adults’ social support and economic conditions. The proportion of low social support (42.6%) and poor economic conditions (42.9%) was higher among the migrants with twenty-five or more years of duration. The results for the migration distance showed a higher proportion of low social support (55.2%) and poor economic conditions (43.3%) among older adults with immigrant status. The rural-to-rural stream migrants had a higher proportion of low social support (45.2%), and rural-to-urban migrants had a higher proportion of poor economic conditions (43.9%) than the other stream migrants. The age at migration (45–49 years) showed a higher percentage of high social support (24.4%) and high economic conditions (47.5%) than other age at migrants.

### Association of migration pattern with social support and economic status

Table 3 depicts the association of social support with the migration pattern, and the table includes four results of association with two models (unadjusted and adjusted). The result of model-I showed the association between social support and migration by distance. The adjusted result showed that intrastate migrants were more likely to have medium social support [RRR = 1.05 (CI; 1.00,1.09)] than non-migrants older adults with low social support. Additionally, immigrants were less likely to have medium [RRR = 0.56 (CI; 0.47–0.68)] and high [RRR = 0.39 (CI; 0.30–0.50)] social support concerning non-migrants with low social support. The interstate migrants were also less likely to have high [RRR = 0.90 (CI; 0.83–0.98)] social support than non-migrants with low social support. Afterward, the adjusted results of model-II suggest that migration duration was not statistically significant for medium social support, but it was significant with 0–9 years of duration associated with high social support. Migration duration 0–9-year were less likely to have high social support [RRR = 0.89 (CI; 0.80-1.00)] than the migrants since birth. The migration stream (model-III) was significantly associated with older adults with medium and high social support. It showed that rural-to-urban [RRR = 1.12 (CI; 1.05–1.19)] and urban-to-urban [RRR = 1.19 (CI; 1.10 1.29)] streams were more likely to have medium social support than rural-to-rural stream older adults’ migrants. Considering high social support, urban-to-rural and urban-to-urban older adult migrants were more likely to have high social support than rural-to-rural migrants. In the case of age at migration (model-IV), adjusted model results showed that older adults 45–59 years of age at migration were significantly less likely to have [RRR = 0.88 (CI; 0.79–0.99) high social support than non-migrants.

**Table 3:** Multinomial logistic regression for the association of social support with migration pattern, LASI Wave-I (2017-18)

Social Support	Unadjusted		Adjusted			
	RRR	95% CI	RRR	95% CI	RRR	95% CI
**By distance of migrants (Model -I)**						
***Low Social Support*** ^***(BO)***^						
***Medium Social Support***						
Non-migrants®	1	[1.00,1.00]	1	[1.00,1.00]		
Intrastate migrants	0.96*	[0.92,0.99]	1.05*	[1.00,1.09]		
Interstate migrants	1.04	[0.97,1.11]	1.03	[0.96,1.11]		
Immigrants	0.54***	[0.45,0.65]	0.56***	[0.46,0.68]		
***High Social Support***						
Non-migrants®	1	[1.00,1.00]			1	[1.00,1.00]
Intrastate migrants	0.77***	[0.74,0.80]			1.02	[0.97,1.07]
Interstate migrants	0.74***	[0.69,0.80]			0.90*	[0.83,0.98]
Immigrants	0.32***	[0.25,0.41]			0.39***	[0.30,0.50]
**By duration of migration (Model-II)**						
***Low Social Support*** ^***(BO)***^						
***Medium Social Support***						
Since Birth®	1	[1.00,1.00]	1	[1.00,1.00]		
0–9 year	1.10*	[1.00,1.21]	1.03	[0.94,1.14]		
10–24 year	1.14***	[1.07,1.22]	1.03	[0.96,1.11]		
25 + year	0.92***	[0.88,0.95]	1.03	[0.99,1.08]		
***High Social Support***						
Since Birth®	1	[1.00,1.00]			1	[1.00,1.00]
0–9 year	0.97	[0.88,1.07]			0.89*	[0.80,1.00]
10–24 year	1.07	[1.00,1.15]			0.93	[0.86,1.01]
25 + year	0.68***	[0.66,0.71]			1.01	[0.96,1.06]
**By Migration stream (Model-III)**						
***Low Social Support*** ^***(BO)***^						
***Medium Social Support***						
Rural-Rural®	1	[1.00,1.00]	1	[1.00,1.00]		
Rural-Urban	1.22***	[1.15,1.29]	1.12***	[1.05,1.19]		
Urban-Rural	1.29***	[1.11,1.51]	1.13	[0.97,1.32]		
Urban-Urban	1.40***	[1.30,1.50]	1.19***	[1.10,1.29]		
***High Social Support***						
Rural-Rural®	1	[1.00,1.00]			1	[1.00,1.00]
Rural-Urban	1.35***	[1.27,1.44]			1.07	[0.99,1.15]
Urban-Rural	1.76***	[1.50,2.06]			1.24*	[1.05,1.48]
Urban-Urban	1.75***	[1.62,1.89]			1.20***	[1.09,1.31]
**Age at migration (Model-IV)**						
***Low Social Support (BO)***						
***Medium Social Support***						
Non-migrants®	1	[1.00,1.00]	1	[1.00,1.00]		
0–14	0.90***	[0.87,0.94]	1.03	[0.99,1.08]		
15–44	1.20***	[1.13,1.27]	1.05	[0.98,1.12]		
45–59	1	[0.92,1.10]	0.99	[0.90,1.09]		
60 and above	0.87*	[0.76,0.99]	0.97	[0.84,1.12]		
***High Social Support***						
Non-migrants®	1	[1.00,1.00]			1	[1.00,1.00]
0–14	0.65***	[0.62,0.68]			1.00	[0.95,1.05]
15–44	1.15***	[1.08,1.23]			0.98	[0.91,1.06]
45–59	0.85**	[0.77,0.94]			0.88*	[0.79,0.99]
60 and above	0.70***	[0.60,0.81]			0.92	[0.78,1.09]

Note: BO = Base outcome & ® = Reference category, *** *p* = < 0.001, **p = < 0.05, & * *p* = < 0.1, age, sex, residence#, marital status, caste, religion, education, working status, MPCE quintile, living arrangement, regions were the control variables # indicate that place of residence was not included in model-III due to collinearity

Table 4 represents the association of economic conditions with migration patterns. It includes four results of association, unadjusted and adjusted. The results of model-I showed the association between economic status and migration by distance. Adjusted results showed that intrastate migrants were more likely to have [RRR = 1.07 (CI; 1.02, 1.120)] middle economic conditions than non-migrants. In addition to the results, intrastate, interstate, and immigrants were 17%, 13%, and 41% more likely to have rich economic conditions than non-migrants with poor economic conditions. The migration duration (model-II) was statistically significant and positively associated with medium and rich economic conditions. The results showed that migrants with 0–9 years and 10–24 years of duration were 33% and 18% more likely to have middle economic conditions, respectively, than migrants since birth. For the rich economic conditions, 0–9 years, 10–24 years, and 25 + years of duration were more likely to have [RRR = 1.66 (CI; 1.50–1.84)], [RRR = 1.29 (CI; 1.20–1.38] and [RRR = 1.10 (CI; 1.05–1.15)] high social support than migrants since births. Finally, the migration stream urban-to-rural was more likely to have middle [RRR = 1.50 (CI; 1.24–1.80)] and rich [RRR = 1.42 (CI; 1.21–1.67)] economic conditions, respectively, concerning rural-to-rural stream but migration stream rural-to-urban and urban to urban were less likely to have middle [RRR = 0.72 (CI; 0.67–0.78)] and rich economic conditions [RRR = 0.48 (CI; 0.45–0.52)], respectively than rural-to-rural migrants. The results of model-IV showed that the pre-retirement age at migration (45–59) was more likely to have middle [RRR = 1.28 (CI; 1.14–1.43)] and rich [RRR = 1.55 (CI; 1.10–1.48)] economic conditions than individuals in the working age and other age at migration, in comparison to non-migrants.

**Table 4:** Multinomial logistic regression for the association of Economic Status with migration pattern, LASI Wave-I (2017-18)

Economic condition	Unadjusted		Adjusted			
	RRR	95% CI	RRR	95% CI	RRR	95% CI
**By distance of migration (Model-I)**						
***Poor (Base outcome)***						
***Middle***						
Non-migrants®	1	[1.00,1.00]	1	[1.00,1.00]		
Intrastate migrants	1.07**	[1.02,1.12]	1.07**	[1.02,1.12]		
Interstate migrants	1.17***	[1.08,1.26]	1.06	[0.97,1.16]		
Immigrants	1.15	[0.91,1.44]	1.24	[0.98,1.56]		
***Rich***						
Non-migrants®	1	[1.00,1.00]			1	[1.00,1.00]
Intrastate migrants	1.16***	[1.12,1.20]			1.17***	[1.12,1.22]
Interstate migrants	1.35***	[1.26,1.44]			1.13**	[1.05,1.22]
Immigrants	1.15	[0.95,1.39]			1.41**	[1.14,1.73]
**By duration of migration (Model-II)**						
*Poor (Base outcome)*						
Middle						
Since Birth®	1	[1.00,1.00]	1	[1.00,1.00]		
0–9 year	1.43***	[1.27,1.60]	1.33***	[1.18,1.49]		
10–24 year	1.28***	[1.19,1.39]	1.18***	[1.08,1.28]		
25 + year	1.03	[0.99,1.08]	1.03	[0.98,1.09]		
***Rich***						
Since Birth®	1	[1.00,1.00]			1	[1.00,1.00]
0–9 year	1.98***	[1.81,2.17]			1.66***	[1.50,1.84]
10–24 year	1.59***	[1.49,1.69]			1.29***	[1.20,1.38]
25 + year	1.06**	[1.02,1.10]			1.10***	[1.05,1.15]
**By the stream of migration (Model-III)**						
***Poor (Base outcome)***						
***Middle***						
Rural-Rural®	1	[1.00,1.00]	1	[1.00,1.00]		
Rural-Urban	0.96	[0.90,1.03]	0.72***	[0.67,0.78]		
Urban-Rural	1.77***	[1.47,2.13]	1.50***	[1.24,1.80]		
Urban-Urban	1.20***	[1.10,1.30]	0.77***	[0.70,0.85]		
***Rich***						
Rural-Rural®	1	[1.00,1.00]			1	[1.00,1.00]
Rural-Urban	0.89***	[0.84,0.94]			0.48***	[0.45,0.52]
Urban-Rural	2.05***	[1.76,2.39]			1.42***	[1.21,1.67]
Urban-Urban	1.44***	[1.35,1.54]			0.57***	[0.52,0.62]
**Age at migration (Model-IV)**						
***Poor (Base outcome)***						
***Middle***						
Non-migrants®	1	[1.00,1.00]	1	[1.00,1.00]		
0–14	1.01	[0.97,1.06]	1.03	[0.97,1.08]		
15–44	1.26***	[1.17,1.35]	1.15***	[1.07,1.24]		
45–59	1.37***	[1.22,1.53]	1.28***	[1.14,1.43]		
60 and above	1.22*	[1.04,1.44]	1.18	[0.99,1.39]		
***Rich***						
Non-migrants®	1	[1.00,1.00]			1	[1.00,1.00]
0–14	1.01	[0.98,1.05]			1.08**	[1.03,1.13]
15–44	1.62***	[1.54,1.72]			1.33***	[1.25,1.42]
45–59	1.80***	[1.65,1.96]			1.55***	[1.40,1.70]
60 and above	1.40***	[1.22,1.59]			1.28**	[1.10,1.48]

Note: ®=Reference category, *** *p* = < 0.001, **p = < 0.05, & * *p* = < 0.1, age, sex, residence#, marital status, caste, religion, education, working status, social support, living arrangement, regions were the control variables # indicate that place of residence was not included in model-III due to collinearity

## Discussion

This study examines the social support and economic conditions among older migrants at destination places. First, examine the distribution of older migrants in India, and second, examine the association of socioeconomic status with migration patterns. More than half of the older adults in India have migrant characteristics; more than 95% of older adults migrated ten or more years before and grew older at their destination. The migrants’ long duration at their destination shows that most (90%) migrated at a relatively early age, before 45, and are growing older at their destination. Primarily in India, child migration occurs due to parents or family-related moves, while adult migration stems from factors such as marriage, education, and employment [30, 41].

Rural-to-rural migration is the dominant stream, followed by rural-to-urban. The proportion of females was higher than the male migrants in the rural-to-rural stream due to primarily marriage-related migration [41–43]. Male migration of this stream appears to result from their migration from areas with low agricultural productivity to regions with new developmental activities in other rural areas [44]. Short-distance (Intra-state) migration is more predominant among older migrants than long-distance migration, such as interstate and international (immigrants) in India. The Indian migration pattern indicates that intrastate migration holds a higher share of total migration among all age groups [37, 38, 45]. So, we argued how migration status influences social support and economic conditions at the place of destination. Does migration pay off in later life? This multinomial analysis has been performed, and the result shows that the migration status is associated with outcome variables. Social support is associated with migration status and other socioeconomic factors. In both unadjusted and adjusted analyses, those with interstate and international migration were less likely to have high social support. Many quantitative studies show that older migrants are, on average, lonelier than their native-born [46, 47]. So, the results of this study conclude that long-distance migration status is negatively associated with social support in later life [48]. The social support of older migrants is shown to vary greatly, depending not only on individual characteristics (e.g., age, educational level, labour market participation, cast, religiosity, region, and length of stay in the country of destination) but also on environmental circumstances and many possible reasons could be for less social support in long-distance migration, such as unknown places, communication barriers to connecting to unfamiliar society, engaging in stressful and harmful conditions, and lack of time to communicate with people due to workload [24].

The duration and stream of migration also play a role in less social support among older migrants at destination places; the new migrants have less social support than non-migrants, but with the increase in migration duration, these differences are reduced []. The economic condition association results with migration show that those with migration status have more chances of being rich than those with non-migration status in both adjusted and unadjusted analyses. Moreover, it is positively associated with economic conditions, which means migration enhances economic status at destination places [30]. Migrants usually move to those countries and within countries that maximize their well-being in terms of their economic condition—mostly from less to more developed countries or places [49,50], and at the reference point of many migrants’ actions is the improvement of their living conditions, the main benefits of migration are the expected earnings at the place of destination [51]. Some of the studies in India show that migration is more influenced by pull rather than push factors [38,41,52], and these pull factors include work-employment opportunities and higher wage differences. A previous study from India showed that migrants experience faster economic growth than non-migrants at the destination, mainly among those migrating from rural to urban streams. Most migrants are self-employed or work as casual workers [53]. With the increase in migration distance, the chance of being more likely to be rich was found in this study and significantly reduced with migration duration; migrants of long-distance and new migrants have richer conditions than those of less distant and settled migrants [30], respectively. Long-distance migrants, including immigrants and interstate migrants, were found to be more wealthy than short-distance migrants as compared to non-migrants. The result supports that long-distance migration enhances economic betterment [29,53,54], so immigrants are more affluent than non-migrants but are not necessarily more likely to have middle economic conditions than non-migrants.

The migrants with a shorter (0–9 years) migration duration show rich economic conditions. This supports the idea that new migrants are healthy and productive at work. However, their health and productivity gradually decrease after spending more time and continually working at their destination [54, 55]. While the study of adult migration shows rural to urban migrants have better economic status [53], this study shows that migrants from urban to rural are more likely to be richer than other streams. The age at migration was also found to be significantly associated with economic conditions in later life. The migrants who migrated during their pre-retirement and working age are likelier to have middle and rich economic conditions than the non-migrants. The overall findings show a clear advantage in economic conditions for older migrants compared to those who stay at their native place in India. So, migration positively affects the economic condition and negatively affects the social support of older migrants in India. The study concludes that the social support and economic conditions among older persons in India differ by their migration status, and these differences also vary with migration patterns, such as migration distance, duration, stream, and age at migration.

This study has some limitations. The migration questions do not include the reasons for migration and do not give answers to social and economic conditions at the time of migration. The present study did not expose multiple natures of migration and migration distance measured in terms of administrative boundary, leading to some bias. Economic conditions measured in this study only in terms of monthly per capita expenditure, which does not provide comprehensive knowledge about economic status; this could also lead to bias. Despite the above limitations, the study has some noteworthy strengths, too. The study findings are based on a large-scale, nationally representative survey of the Indian older adult population, and study findings can be generalized at the national level. Moreover, the data provides the current estimates as the data were collected recently and released in 2021. Furthermore, the studies must examine the social and economic status among return migrants and the left-behind population.

## Conclusion

The findings of this study suggest that more than half of the older adults were migrants, with 8 out of 10 and 9 out of 10 were migrated before 25 years and within states, respectively. Findings suggest that migration positively affects the expenditure status and negatively affects the social support of older migrants in India. Additionally, long-distance and short-duration migration has a negative association with social support and a positive association with the economic status of older adult migrants in later life. A better understanding of the older migration pattern and its consequences on the ageing process is crucial for governments and policymakers to reduce the challenge of older migrants in health care and social welfare systems. Older migrants must be focused in the study on the development perspectives to achieve active and healthy ageing and to achieve the goal of leaving no one behind. Whether migrants or not, everyone should be focused on development studies. Researchers must concentrate on migration and its impact from an ageing perspective regarding their social and economic development. In exploring migration’s effect on social and economic status, policymakers should prioritize migrants in their agenda to maintain the socioeconomic status of older migrants in India to achieve the sustainable goal of active and healthy ageing for all.

## Data Availability

The study utilizes a secondary source of data that is freely available in the public domain through a request form (LASI Wave 1 Data Request form (www.iipsindia.ac.in).

## Abbreviations

LASI: Longitudinal Ageing Study in India
MPCE: Monthly Per Capita Expenditure
POLR: Place of Last Residence
RRR: Relative Risk Ratio
WHO: World Health Organization
